# The impact of acupuncture on neuroplasticity after ischemic stroke: a literature review and perspectives

**DOI:** 10.3389/fncel.2022.817732

**Published:** 2022-11-10

**Authors:** Siru Qin, Zichen Zhang, Yadan Zhao, Jingyi Liu, Jiwen Qiu, Yinan Gong, Wen Fan, Yongming Guo, Yi Guo, Zhifang Xu, Yang Guo

**Affiliations:** ^1^Research Center of Experimental Acupuncture Science, Tianjin University of Traditional Chinese Medicine, Tianjin, China; ^2^School of Acupuncture & Moxibustion and Tuina, Tianjin University of Traditional Chinese Medicine, Tianjin, China; ^3^National Clinical Research Center for Chinese Medicine Acupuncture and Moxibustion, Tianjin, China; ^4^Department of Rehabilitation Physical Therapy Course, Faculty of Health Science, Suzuka University of Medical Science, Suzuka, Japan; ^5^School of Traditional Chinese Medicine, Tianjin University of Traditional Chinese Medicine, Tianjin, China; ^6^Acupuncture Department, First Teaching Hospital of Tianjin University of Traditional Chinese Medicine, Tianjin, China

**Keywords:** acupuncture, ischemic stroke, neuroplasticity, neurogenesis, axon regeneration, synapse, neurotrophic factors, glia

## Abstract

Ischemic stroke is common in the elderly, and is one of the main causes of long-term disability worldwide. After ischemic stroke, spontaneous recovery and functional reconstruction take place. These processes are possible thanks to neuroplasticity, which involves neurogenesis, synaptogenesis, and angiogenesis. However, the repair of ischemic damage is not complete, and neurological deficits develop eventually. The WHO recommends acupuncture as an alternative and complementary method for the treatment of stroke. Moreover, clinical and experimental evidence has documented the potential of acupuncture to ameliorate ischemic stroke-induced neurological deficits, particularly sequelae such as dyskinesia, spasticity, cognitive impairment, and dysphagia. These effects are related to the ability of acupuncture to promote spontaneous neuroplasticity after ischemic stroke. Specifically, acupuncture can stimulate neurogenesis, activate axonal regeneration and sprouting, and improve the structure and function of synapses. These processes modify the neural network and function of the damaged brain area, producing the improvement of various skills and adaptability. Astrocytes and microglia may be involved in the regulation of neuroplasticity by acupuncture, such as by the production and release of a variety of neurotrophic factors, including brain-derived neurotrophic factor (BDNF) and nerve growth factor (NGF). Moreover, the evidence presented indicates that acupuncture promotes neuroplasticity by modulating the functional reconstruction of the whole brain after ischemia. Therefore, the promotion of neuroplasticity is expected to become a new target for acupuncture in the treatment of neurological deficits after ischemic stroke, and research into the mechanisms responsible for these actions will be of significant clinical value.

## Introduction

Ischemic stroke is common in the elderly, and its incidence increases with age. It is one of the leading causes of long-term disability worldwide (Go et al., 2014). The primary cause of ischemic stroke is the interruption of blood flow by cerebral vascular occlusion. Acutely and sub-acutely lack of blood supply produces metabolic acidosis, excitotoxicity, inflammation, oxidative stress, and cytotoxic edema, resulting in necrosis or apoptosis of neurons. However, spontaneous recovery appears in the brain damaged by an ischemic stroke. For instance, in the subacute stage of stroke, neurotrophic factors (NTFs) and associated signaling pathways, such as protein-serine-threonine kinase (Akt) pathway, trigger the neuroprotective effect. In the chronic phase, neurogenesis, angiogenesis, and synaptogenesis are the main processes of spontaneous neuroplasticity, but the repair is often incomplete due to the limited regenerative capacity of neurons (Chavez et al., 2017). Therefore, understanding the mechanisms of promoting spontaneous and therapeutic neuroplasticity-mediated structural reconstruction and functional recovery is of great significance for the treatment and prognosis of ischemic stroke (Cassidy and Cramer, 2017).

Brain neuroplasticity refers to the ability of the brain to modify its morphological structure and functional activity in response to internal or external stimuli (Carey et al., 2019). It occurs throughout the entire lifespan, and the structural and functional plasticity may be enhanced after brain injury. Brain neuroplasticity can be observed at different levels, such as changes in the structure and function of synapses, cells, and regions, subsequent modifications in the communication and networks among cells and regions, and, ultimately, changes in neurologic behavior and function, such as sensory perception, motor behavior, and cognition (Murphy and Corbett, 2009; Pekna et al., 2012). Since the brain has a remarkable capacity for plasticity and reorganization, it would be useful to exploit these properties to improve the clinical efficacy of ischemic stroke therapy.

Acupuncture, a process of inserting fine needles into the skin or deep tissues of specific parts (acupoints) of the body, is an essential component of Traditional Chinese Medicine. Acupuncture can be applied by hand, electric stimulation, or heating (Li and Wang, 2013), and is characterized by a safe, efficient, economical, and simple operation. Lines of clinical and experimental evidence have demonstrated that acupuncture can improve ischemic stroke-induced neurological deficits, especially for the sequelae of stroke (World Health Organization, 2002; Li et al., 2021; Shao et al., 2021). The effect of acupuncture starts from the stimulation of the acupoints, i.e., excitable complexes of muscle and skin nerves with a high density of nerve endings (Li et al., 2004). After converting physical or chemical information to electrical activity in acupoints, the signal is sent along afferent fibers to the spinal cord and brain (Zhao, 2008). There is evidence that acupuncture can modulate neuroplasticity in central nervous system (CNS) by modifying neural structure and function (Chavez et al., 2017). However, the detailed mechanisms of the effect of acupuncture on brain neuroplasticity in ischemic stroke have not been systematically reviewed.

This review analyzes neuroplasticity as the basis for the discussion of the effects of acupuncture on ischemic stroke and their potential mechanisms. The understanding of how acupuncture promotes and modulates neurogenesis, axonal regeneration and sprouting, synaptic plasticity, neuroglial crosstalk, and functional reconstruction reviewed in this work provides novel insights into the therapeutic mechanism of this procedure on ischemic stroke. It is hoped that the information provided will promote the clinical application of acupuncture for the functional recovery of ischemic stroke worldwide.

## Methods

### Search strategy

We searched the PubMed database for studies published between January 2010 and April 2021 using the MeSH terms “Acupuncture,” “Ischemic Stroke” and relevant entry terms. The search identified 211 relevant articles.

#### Study selection

**Figure 1:**
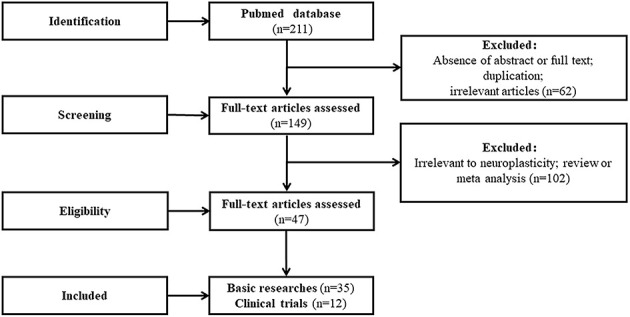
A flowchart of this search process.

We developed the following study inclusion criteria to study the treatment of ischemic stroke by acupoint stimulation *via* regulating neuroplasticity: stimulation methods included manual acupuncture, electroacupuncture, and moxibustion, and the research topics were related to neuroplasticity, specifically, neurogenesis, axon sprouting, axon regeneration, synaptic plasticity, and functional reorganization of the brain regions. Following the identification of 211 articles by the search engine, we performed a manual search of the reference lists of articles to identify further relevant articles that met the inclusion criteria based on the titles and abstracts. We excluded 62 articles due to duplication, the lack of an abstract or lack of relevance to ischemic stroke before conducting full-text assessments. This left 149 articles, including 66 basic research articles, 54 clinical research articles, and 29 review articles or meta-analyses. The full texts of the 66 basic research and 54 clinical research articles were obtained and evaluated carefully. Of the 66 basic research articles, 35 were selected and 31 were excluded as they did not focus on neuroplasticity. Of the 54 clinical studies, 12 were selected and 42 were excluded as they described single case reports, editorials, incomplete or uncontrolled trials, or were irrelevant to neuroplasticity. Thus, a total of 47 articles were included in our review. A flowchart of this search process is illustrated in Figure 1.

#### Data extraction

Two authors independently evaluated the titles and abstracts of the retrieved articles and assessed the full texts of the articles. All of the 47 articles were finally included in the review, from which the data were extracted and listed according to the prespecified criteria, to analyze the mechanisms of acupuncture in regulating neuroplasticity in the treatment of ischemic stroke. Any disagreement was resolved by discussion between the authors.

### Clinical Effect of Acupuncture on Ischemic Stroke

Neurological deficits after ischemic stroke can be self-repaired to varying degrees, which may be due to the plasticity of the remaining brain tissue. Despite this, damage to the functional areas of the brain may persist in areas responsible for motion, cognition, sensation, vision, and language (Rathore et al., 2002). Therefore, a wide range of rehabilitation treatments for stroke recovery has been developed, including the use of drugs, stem cells, behavioral therapy, robotics, and acupuncture (Cramer, 2008). Of these types of therapy, many systematic reviews have indicated that acupuncture can improve the neurological function of patients with ischemic stroke (Liu et al., 2015; Yang et al., 2016; Hung et al., 2019). A Cochrane review included 31 trials with a total of 2,257 participants in the subacute or chronic stages of stroke, and found that compared with no acupuncture, acupuncture was beneficial for the improvement of dependency, global neurological deficiency, and specific neurological impairments including motor, cognitive, and swallowing functions, as well as depression and pain (Yang et al., 2016). Four trials in this systematic review and other randomized controlled trials (RCTs) used the Fugl-Meyer Assessment scale (FMI) to evaluate the impact of acupuncture therapy after an ischemic stroke on the motor function of the upper and lower limbs. The results showed greater improvement in motor function in the acupuncture group compared with the non-acupuncture group (Li et al., 2015; Yang et al., 2016; Wu J. et al., 2017). The results of 11 trials in this review and other following RCTs found that the dependence measured by continuous scales [Barthel Index (BI), Modified Barthel Index and Activity of Daily Living Scale] in the acupuncture group was significantly improved compared with the control group (Yang et al., 2016; Wu J. et al., 2017; Wu et al., 2018). Furthermore, in this review, 13 trials were conducted to evaluate the improvement of cognitive function by acupuncture, using the Mini-Mental State Examination, Montreal Cognitive Assessment Scale, and Revised Hasegawa Dementia Scale. The results showed that acupuncture was superior to non-acupuncture in the improvement of cognitive function in convalescent stroke patients. The authors also concluded that acupuncture is most beneficial in the first 3 months with reduced benefit after 6 months. However, in terms of long-term follow-up, including mortality and quality of life, there is a lack of data from RCTs on the clinical outcomes of acupuncture in the treatment of stroke (Yang et al., 2016). The analysis of the recurrence of ischemic stroke treated with or without acupuncture has shown that patients treated with both medications and acupuncture had a lower rate of recurrence than patients treated only pharmaceutically (Shih et al., 2015). However, we still need more reliable evidence of major outcomes from more stringent RCTs, including the setting of grouping concealment, to draw conclusions about the efficacy of acupuncture on stroke.

RCTs focused on the use of acupuncture for the treatment of stroke have shown satisfactory results when acupuncture is applied at acupoints to treat various stroke sequelae, such as hemiplegia, dysphagia, and cognitive impairment. For hemiplegia, the most commonly used acupoints include *Baihui* (GV20), *Yintang* (EX-HN3), and the anterior oblique line of vertex-temporal of the head, *Jianyu* (LI15), *Quchi* (LI11), *Shousanli* (LI10), *Waiguan* (TB5), *Hegu* (LI4) of upper limbs, *Liangqiu* (ST34), *Zusanli* (ST36), *Yanglingquan* (GB34), *Sanyinjiao* (SP6), *Fenglong* (ST40), *Jiexi* (ST41), and *Taichong* (LR3) of lower limbs. These acupoints are important to inhibit muscle spasm, restore the coordination function of extensors and flexors, and inhibit their synergic movement, improving in this manner the limb function in hemiplegia (Chen J. et al., 2014; Wang et al., 2017). For aphasia, No. 1, 2, and 3 language sections of the head are selected according to the type of aphasia, in which language section No. 1 is for motor aphasia, No. 2 is for anomic aphasia, No. 3 is for sensory aphasia, and No. 1 combined with No. 2 is for mixed aphasia. In addition, *Fengchi* (GB20), *Yamen* (GV15), *Jinjin* (EX-HN12), *Yuye* (EX-HN13), *Tongli* (HT5), and* Lianquan* (CV23) acupoints are used, which are located near the tongue and above the Heart Meridian of Hand-shaoyin associated with the tongue (Sun et al., 2012). For a mild cognitive impairment, GV20, *Shenting* (GV24), *Benshen* (GB13), *Taiyang* (EX-HN5), *Touwei* (ST8), *Sishencong* (EX-HN1) are employed. Among them, EX-HN5, ST8, and EX-HN1 acupoints are located in the anterior and medial sides of the temporal lobe and adjacent areas, which control memory function and mental activity (Chen J. et al., 2014; Kalaria et al., 2016). Although acupuncture protocols differ according to the different impaired functions, they all conform to the principle of the of integral-local combined selection, i.e., matching the acupoints of brain functional areas with those of specific lesion areas.

### Acupuncture Modulates Neuroplasticity

Recovery of lost functions after ischemic stroke is thought to depend on neuroplasticity, that is, the ability of the brain to restructure and reconstruct in response to endogenous and exogenous stress and injuries (Murphy, 2015; Dąbrowski et al., 2019). Neuroplasticity manifests in short-term functional changes and long-term structural changes. Short-term functional changes consist of modifications of synaptic efficiency, while the long-term structural changes reflect adaptations of neural connections. The core components of neuroplasticity are neurogenesis, axon sprouting, axon regeneration, and synaptic plasticity (Hickmott and Ethell, 2006; Lillard and Erisir, 2011). These processes and the potential mechanisms by which acupuncture can affect them are reviewed below and summarized in Table 1.

**Table 1:** The mechanisms of acupuncture on regulating neuroplasticity in the treatment of ischemic stroke.

**Study**	**Model/Objects**	**Intervention**	**Acupoints**	**Comparison**	**Acupuncture parameters**	**Effect measurements**	**Biochemical measurements**
Luo et al. (2014)	MCAO rats	MA	GV26	Non-MA	Thrust/lifted at 3 times per second, 1 min, 3 days after MCAO immediately	Zausinger’ 6-point scale neural deficit scores↑; cerebral blood flow↑	Cortex and hippocampus: BrdU^+^ cells↑, nestin^+^ cells↑, BrdU^+^ /nestin^+^ cells↑, nestin mRNA↑; Cortex, hippocampus, and ST: GSK-3β↑, PP2A↓, GSK-3β/PP2A↑
Tao et al. (2010)	MCAO rats	EA	ST36, LI11	Non-EA	1/20 Hz, 20 min, 4/7/14/21 days from 24 h after MCAO	Neurological deficit scores↓	SVZ: BrdU^+^ cells↑, BrdU^+^ /GFAP^+^ cells↑, BrdU^+^ /NeuN^+^ cells↑
Liao et al. (2017)	MCAO rats	EA	ST36, ST37	Non-EA	EA1: 2 Hz EA2: 15 Hz	mNSS↓; rotarod test: latency time↑; I/H ratio (EA1)↑	Penumbra area: Ki67^+^ cells (EA1)↑, GFAP^+^ cells (EA1)↑, nestin^+^ cells↑; ischemic core area: nestin^+^ cells↑
Tan et al. (2018)	MCAO rats	EA	GV14, GV20	Non-EA	5/20 Hz, 2-4 mA, 30 min	mNSS↓	CA1 region: viable neuron percentage↑; DG zone: BrdU^+^ /nestin^+^ cells↑, BrdU^+^ /DCX^+^ cells↑; brain: PRG5↑, RhoA↓, LPA↓, NogoA↓
Kim et al. (2014)	MCAO mice	EA	GV14, GV20	Non-EA	2 Hz, 20 min, 10 days from 5 d after MCAO	Rotarod test: latency time↑; MWM test: mean time to find platform↓	Ipsilateral and contralateral hemisphere: BrdU^+^ cells↑; whole brain: BrdU^+^ /DCX^+^ cells↑, BrdU^+^ /NeuN^+^ cells↑; ipsilateral hippocampus, SVZ and cortex: BrdU^+^ cells↑; ipsilateral hippocampus and SVZ: BrdU^+^ /DCX^+^ cells↑, BrdU^+^ /NeuN^+^ ↑, BrdU^+^ /GFAP^+^ cells↑; ipsilateral hemisphere: BDNF and VEGF mRNA↑; ipsilateral hippocampus: mBDNF↑; ipsilateral hippocampus and cortex: VEGF↑; ipsilateral hippocampus and SVZ: mBDNF^+^ cells↑; hippocampus and ipsilateral SVZ: VEGF^+^ cells↑; ipsilateral and contralateral hippocampus, SVZ and cortex: pPI3K^+^ /BrdU^+^ cells↑
		EA	GV14, GV20	Non-EA	2 Hz, 20 min, 12 days from 5 d after MCAO	Corner test: number of turns↓; cylinder test: held on cylinder wall with both paws↓; passive avoidance test: entry latency↑	ST: atrophic changes↑; ST and SVZ: BrdU^+^ cells↑, Ki67^+^ /Sox2^+^ cells↑, mBDNF^+^ /NeuN^+^ cells↑, pTrkB↑, pCREB↑; ST, SVZ and hippocampus: Ki67^+^ cells↑; ST, SVZ and hippocampus: mBDNF↑; hippocampus: NT4↑, NT4^+^ /NeuN^+^ cells↑
Kim et al. (2018)	MCAO mice	EA+mBMSC	GV14, GV20	mBMSC	2 Hz, 20 min, 12 days from 5 d after MCAO	Corner test: number of turns↓; cylinder test: held on cylinder wall with both paws↑; passive avoidance test: entry latency↑	ST and SVZ: BrdU^+^ cells↑, BrdU^+^ /DCX^+^ cells↑, Ki67^+^ /PSA-NCAM^+^ cells↑, NT4^+^ /NeuN^+^ cells↑, pCREB↑, pCREB^+^ /DCX^+^ cells↑; ST, SVZ and hippocampus: Ki67^+^ cells↑, Ki67^+^ /Sox2^+^ cells↑, mBDNF↑, NT4↑; hippocampus: mBDNF^+^ /NeuN^+^ cells↑
Chen et al. (2015)	MCAO-induced focal I/R injury rats	EA	ST36, LI11	Non-EA	1/20 Hz, 30 min, 3 days from 24 h after MCAO	Neurological deficits scores↓; infarct volume↓	Cortical peri-infarct area: GFAP^+^ reactive astrocytes↑, nestin^+^ cells↑, nestin^+^ /GFAP^+^ cells↑, Wnt1 and β-catenin↑, transcription of GSK3↓
Zhao et al. (2013)	MCAO rats	EA	GV20, GV26	Non-EA	4/20 Hz, 1-2 mA, 15 min, 3/7/14/21 days from 3 d after MCAO	Total mNSS↓, movement score of mNSS↓; MWM test: escape latency↓	DG zone: BrdU^+^ /GFAP^+^ cells↑, BrdU^+^ /NeuN^+^ cells↑, Notch1 and Hes1↑
Hong et al. (2013)	tMCAO rats	EA	ST36, LI11	Non-EA	5/20 Hz, 2-4 mA, 20 min, 4 weeks	Infarct volume↓; time spent walking, rearing and grooming↑; time spent feeding↓	Raldh1 and Raldh2 mRNA↑
		EA	TE5, ST36	Non-EA	20 Hz, 1 mA, 30 min, 7 days from 24 h after MCAO	mNSS↓; infarct volume↓	Ischemic cortical region: histological changes↓; SVZ and hippocampal: apoptotic cells↓, miR-223↑, PTEN mRNA↓, nestin mRNA↑, Notch1 protein and the NOTCH1 gene↑
Sha et al. (2019)	MCAO-induced focal I/R injury rats	EA+AntagomiR-223-3p	TE5, ST36	EA	20 Hz, 1 mA, 30 min, 7 days from 24 h after MCAO	mNSS↑	Ischemic cortical region: histological changes↑; SVZ and hippocampal: apoptotic cells↑, miR-223↓, PTEN mRNA↑, nestin mRNA↓
Zhang et al. (2020)	MCAO rats	EA	ST36, LI11	Non-EA	1/20 Hz, 1 mA, 30 min, 21 days	mNSS↓	Peri-ischemic ST: CD81 and TSG101↑, exosomal miR-146b↑, miR-146b↑; peri-ischemic ST and SVZ of the ischemic hemisphere: NeuN^+^ /Brdu^+^ cells↑; SVZ of the ischemic hemisphere: NeuroD1↑, NeuroD1^+^ /DCX^+^ cells↑
		EA+miR-146b inhibitors	ST36, LI11	EA	1/20 Hz, 1 mA, 30 min, 21 days	-	Peri-ischemic ST and SVZ of the ischemic hemisphere: NeuN^+^ /Brdu^+^ cells↓; SVZ of the ischemic hemisphere: NeuroD1↓, NeuroD1^+^ /DCX^+^ cells↓
Zhou et al. (2011)	MCAO rats	EA	ST36, PC6	Non-EA	4/20 Hz, 0.5 mA, 20 min, 1/7/14 days from 12 h after MCAO	Bederson neurologic deficits scores↓	ST and cortical peri-infarct area: GAP-43^+^ cells↑
Xu et al. (2017)	Heat-coagulation MCAO rats	MA+RT	GV26, PC6	Non-MA+non-RT	GV26: thrust/lifted like sparrow pecking for 10 times, PC6: thrust/lifted and turned, 1 min. 7/ 14/21 days from 3 d after MCAO	Zea-Longa 5-point assessment: neurological deficits scores↓; balance beam test scores↓; rotarod test scores↓	Cortical peri-infarct area: GAP-43^+^ cells↑
Qing et al. (2016)	MCAO rats	Group1: bilateral EA+RT, group2: unilateral EA+RT	ST36, GV20, LI11	Non-EA+non-RT	5/10 Hz, 2 mA, 30 min, 6 days/week, 2 weeks from 24 h after MCAO	Neurological deficits scores↓	Ischemic frontal cortex: the arrangement of nerve cells in slightly disordered, some nuclei were pyknotic, the damage of cells↓; hippocampal CA3 region: GAP-43↑, SYP↑
Chen S. Q. et al. (2020)	MCAO-induced transient cerebral I/R injury model	EA	GV20	Non-EA	2 Hz, 1 mA, 30 min, 7 days after reperfusion	Infarct size↓; Garcia JH neurological score↑	Ischemic penumbra cortex: OMgp↓, NogoA↓, NgR↓, NgR mRNA↓, ROCK2↓, MYPT1↓, MLC1↓, RhoA↓, ROCK mRNA↓, MLC mRNA↓, GAP43↑, BDNF↑, GAP43 mRNA↑, BDNF mRNA↑
		EA	GV20	Non-EA	2/10 Hz, 1-2mA, 30 min, 5 days from 24 h after MCAO	Infarct volumes↓; Garcia JH neurological score↑; time on rotarod↑; limb placement test scores↑; left/total body swing numbers↓; unsuccessful contralateral forelimb placing↓	Ischemic penumbra: miR-132↑, SOX2↓
Zhao et al. (2018)	MCAO-induced focal I/R injury rats	EA+miR-132 inhibitor	GV20	EA	2/10 Hz, 1-2 mA, 30 min, 5 days from 24 h after MCAO	Infarct volumes↑; Garcia JH neurological score↓; time on rotarod↓; limb placement test scores↓; left/total body swing numbers↑; unsuccessful contralateral forelimb placing↑	Ischemic penumbra: SOX2↑, miR-132↓
		EA	GV20	Non-EA	2/10 Hz, 1-2 mA, 30 min, 5 days/week, 4 weeks	mNSS↓; time on rotarod↑; grip strength test: newtons↑	Middle and left sides of the spinal cord gray matter (C3-5): BDA^+^ CST axons amounts↑; injection ipsilateral site: fiber numbers↑; ischemic penumbra: NF-200↑, RhoA↓, GAP43 protein↑, pirb mRNA↓, PirB protein↓, PirB^+^ neurons↓, miR-181b↑
Deng et al. (2016)	MCAO rats	EA+miR-181b inhibitor	GV20	EA	2/10 Hz, 1-2 mA, 30 min, 5 days/week, 4 weeks	mNSS↑; time on rotarod↓; grip strength test: newtons↓	Ischemic penumbra: pirb mRNA↓, PirB protein↓
Yi et al. (2006)	Heat-coagulation MCAO rats	EA1: 2 weeks after ischemia, EA2: 5 weeks after ischemia	GV14, GV20	Non-EA	EA1: 5/10 Hz, 30 min, 2 weeks EA2: 5/10 Hz, 30 min, 5 weeks	-	Ischemic cortex: Nv↓, Sv↑, Vv↓, the curvature of synaptic interface (EA2)↓, PSD (EA2)↑, synaptic cleft width↑; cortical peri‑infarct area: COD of P38 (EA2)↑, COD of GAP-43↑, NGF^+^ cells↑, BDNF^+^ cells↑
Xia et al. (2017)	MCAO rats	MA	KI3, LR3	Non-EA	Perpendicularly needled 2–3 mm in depth, 30 min, 7 days/course, 2 courses	mNSS↓; MWM test: escape latency↓	Hippocampal regions: BDNF, SYN, PSD and synaptic curvatures↑, synapse cleft width↓
Xie et al. (2019)	MCAO-induced I/R injury rats	EA	GV20, GV24	Non-EA	1/20 Hz, 0.2 mA, 30 min, 14 days from 24 h after MCAO/R	Zea-Longa 5-point assessment: neurological deficits scores↓; step-down passive avoidance test: the step-down latency time↑	Hippocampal CA1 region: PSD-95^+^ and SYN^+^ cells↑, synapses numbers↑, fusion of synaptic space↓, loss of synaptic vesicles↓, incomplete synaptic structure↓, swelling of the presynaptic terminal↓, p-JAK2 and p-STAT3↓
Lin et al. (2016)	MCAO-induced I/R injury rats	EA	GV20, GV24	Non-EA	1-20 Hz, 30 min, 6 days from 24 h after reperfusion	Infarct volumes↓; MWM test: escape latency↓, swim distance↓, frequency of crossing the platform↑	Hippocampus: density of dendritic spines↑; hippocampus of the left brain: Cdc42, Rac1 and F-actin↑, RhoA protein↓
Liu et al. (2017)	MCAO-induced cognitive deficit model	EA	GV20, GV24	Non-EA	1-20 Hz, 0.2 mA, 30 min, 14 days from 24 h after MCAO	MWM test: escape latency↓, probe time↑, time spent in target quadrant↑	Left cortex, hippocampus, corpus ST, and thalamus: lesions volumes↓; hippocampal CA1: density of dendritic spines↑, number synapses↑, total LIMK1 level and p-LIMK1↑, miR-134 expression↓
Yang et al. (2017)	Stroke patients (*n* = 10)	MA	LI11, LI10, TB5, LI4, ST36, GB34, SP6, EX-UE9	Self-control	Thrust/lifted at 120 times per min, rotated at 180 degrees, 120 circle per min, conducted until *Deqi*	-	Left FDI: MEPs↓; right FDI: MEPs↑; the percentage of MEP amplitudes from TS at CS intensity 100%, 130% and 150%↑; inhibition from the right M1 (contralateral to acupuncture side) to the left M1 (ipsilateral to acupuncture side)↓
He et al. (2019)	Stroke patients (*n* = 18)	MA	LI11, TB5	Sham acupuncture	Thrust/lifted until *Deqi*	-	Contralateral hemisphere of the acupuncture sites: the cortex excitability (when 12–20min with the needle *in situ*)↓, the MEP amplitudes (when 8–10 min with the needle removed)↑; ipsilateral hemisphere of the acupuncture sites: the MEP amplitudes (when 4–10 min with the needle *in situ*)↑, the cortex excitability (when 1–5 min with the needle removed)↑; contralateral cortical of the acupuncture sites: MEP amplitudes induced by acupoint needling↓, MEP amplitudes after needle removal↑; ipsilateral cortical of the acupuncture sites: MEP amplitudes induced by acupoint needling↓, MEP amplitudes after needle removal↑
		MA+PAS	LI11, TB5	Self-control	Thrust/lifted until *Deqi*	-	Contralateral cortical of the acupuncture sites: MEP amplitudes after PAS intervention and needle removal↑, MEP amplitudes induced by needle *in situ*↓; ipsilateral cortical of the acupuncture sites: MEP amplitudes after PAS intervention with the needle *in situ* and removal↑
		MA+MP	LI11, TB5	Self-control	Thrust/lifted until *Deqi*	-	Contralateral cortical of the acupuncture sites: MEP amplitude after MP and pretreatment with acupuncture↓, MEP amplitudes after the combined treatment with acupuncture and MP↓, MEP amplitudes after needle removal↑, FDI muscle MEP amplitude after acupuncture and acupuncture + MP↑
Lin and Hsieh (2010)	MCAO rats	EA	GV20	Non-EA	2 Hz, 2 mA, 20 min, 10 min after MCAO	Neurological scores↓	Hippocampal CA1: LTP↑, NR1↓, TRPV1↓
Ye et al. (2017)	2VO-induced Vascular dementia rats	MA	Group1: ST36, GV20; Group2: GV20, GV24; Group3: ST36, SP10	-	Twirling reinforcing manipulation, 6 days a week, 2 weeks from 3 days after 2VO	MWM test: escape latency↑, swimming distance↑, time in the target quadrant↑ (group1 was superior over other groups)	-
		MA	ST36, GV20	Non-MA	Twirling reinforcing manipulation, 6 days a week, 2 weeks from 3 days after 2VO	-	Hippocampus: LTP at the PP-DG synapse↑, Dopamine, HVA and epinephrine↑; hippocampal DG region: D1R and D5R↑
		MA+D1/D5Rs Antagonists SCH23390	ST36, GV20	MA	Twirling reinforcing manipulation, 6 days a week, 2 weeks from 3 days after 2VO	MWM test: escape latency↓, swimming distance↓, time in the target quadrant↓	PP-DG synapse: LTP↓

Notes: ↑, upregulated by acupuncture; ↓, downregulated by acupuncture. Abbreviation: MCAO, middle cerebral artery occlusion; MA, manual acupuncture; GV26, Shuigou; GSK-3β, glycogen synthase kinase 3β; PP2A, Protein phosphatase 2A; EA, electroacupuncture; ST36, Zusanli; LI11, Quchi; GFAP, glial fibrillary acidic protein; SVZ, subventricular zone; ST37, Shangjuxu; mNSS, modified Neurological Severity Score; I/H ratio, ratio of the ischemic area to the ipsilateral hemisphere area; GV14, Dazhui; GV20, Baihui; DCX, doublecortin; DG, dentate gyrus; PRG5, plasticity-related gene 5; RhoA, Ras homolog family member A; LPA, Lysophosphatidic acid; NogoA, neurite outgrowth inhibitor protein A; BDNF, brain-derived neurotrophic factor; VEGF, vascular endothelial growth factor; mBDNF, mature BDNF; ST, striatum; pPI3K, phosphorylated phosphatidylinositol 3-kinase; pTrkB, phosphorylated tyrosine receptor kinase B; pCREB, phosphorylated cyclic adenylate monophosphate response element-binding protein; MSCs, mesenchymal stem cells; PSA-NCAM, polysialylated form of the neural cell adhesion molecule; NT4, Neurotrophin 4; I/R, ischemia/reperfusion; tMCAO, transient middle cerebral artery occlusion; Raldh1, retinaldehyde hydroxylase 1; Raldh2, retinaldehyde dehydrogenase 2; TE5, Waiguan; PC6, Neiguan; GAP-43, growth-associated protein 43; RT, rehabilitation training; SYP, synaptophysin; OMgp, oligodendrocyte-myelin glycoprotein; NgR, Nogo receptor; MYPT1, myosin phosphatase target subunit-1; BDA, biotionylated dextran amine; CST, corticospinal tract; PirB, Paired immunoglobulin-like receptor B; Nv, numerical density; Sv, surface density; Vv, volume density; COD, corneal optical density; P38, synaptophysin; KI3, Taixi; LR3, Taichong; SYN, synaptophysin; PSD, post-synaptic density; GV24, Shenting; Cdc42, cell division cycle 42; Rac1, Ras-related C3 botulinum toxin substrate 1; LIMK1, LIM domain kinase; MEPs, motor-evoked potentials; FDI, first dorsal interosseous; TS, testing stimulus; CS, conditioning stimulus; M1, primary motor cortex; PAS, paired-associative stimulation; MP, motor practice; LTP, long-term potentiation; NR1, N-methyl-d-aspartate receptor subtype 1; TRPV1, transient receptor potential vanilloid subtype 1; 2VO, permanent bilateral common carotid artery occlusion; SP10, Xuehai; PP, perforant pathway; HVA, homovanillic acid; D1R, dopamine D1 receptor; D5R, dopamine D5 receptor.

#### Acupuncture promotes endogenous neurogenesis

The neurogenesis of the adult mammalian brain continues throughout the lifespan of an organism. The main neurogenic niches are located in the subventricular zone (SVZ) of the lateral ventricle and the subgranular zone (SGZ) of the dentate gyrus in the hippocampus. Neural stem cells (NSCs) and neural precursor cells (NPCs) from SVZ migrate to the olfactory bulb through the rostral migratory stream and are thought to be responsible for the maintenance and reorganization of the interneuron system within the olfactory bulb. The cells from the SGZ migrate to the granule cell layer and differentiate into granulosa cells that form synapses with existing neurons and have an important function in neuroplasticity (Merson and Bourne, 2014; Xiao et al., 2018). Increasing evidence suggests that adult neurogenesis also occurs in several other regions of the CNS, including the spinal cord, striatum, neocortex, cerebellum, substantia nigra, amygdala, and hypothalamus, particularly following a CNS injury (Martino et al., 2011; Sandvig et al., 2018).

Cell death occurs immediately in the ischemic core of the cerebral infarction, followed by the spread of injury to the ischemic penumbra. Therefore, enhancing the survival, proliferation, and migration of endogenous NSCs to the injured region by upregulating local secretion of NTFs facilitates the formation of new synapses and circuits and the restoration of the structure and function of the brain. NSCs can differentiate into neurons, astrocytes, oligodendrocytes, and other neural cells, which gradually migrate to target areas (Yamashita et al., 2006; Brouns and De Deyn, 2009). Since the number of endogenous NSCs and NPCs is too small to sustain the recovery of neurologic functions after ischemic stroke, strategies aiming at the promotion of their proliferation and directional differentiation into neurons with normal functions are the focus of intense research. At the same time, the transformation of astrocytes into neurons to assume neuronal functions after brain injury is also a new research hotspot (Magnusson et al., 2014).

Many studies have suggested that acupuncture can promote the proliferation, migration, and differentiation of NSCs, protecting the brain from ischemic damage and improving neurological deficits. For instance, the study conducted by Luo et al. (2014) demonstrated that middle cerebral artery occlusion (MCAO) in rats produced a large number of cells positive for nestin (a specific marker of NSCs), 5-bromo-2’-dexoyuridine (BrdU, a marker of cell proliferation), and cells double-positive for both markers in the brain, while cells positive for these markers were absent in control non-MCAO mice, and acupuncture at *Shuigou* (GV26) further increased the number of nestin/BrdU positive regenerative cells in MCAO mice. These results indicated that a certain extent of “self-healing” takes place after brain damage induced by focal cerebral ischemia, and acupuncture treatment promotes the proliferation of NSCs. This study also demonstrated that the expression of nestin mRNA in the cortex in the acupuncture group was much higher than in the non-MCAO and non-acupuncture groups. Moreover, with acupuncture, nestin expression increased slightly in the hippocampus and decreased in the striatum, indicating that acupuncture at GV26 promoted more extensive proliferation and/or migration of new neurons to the cortex than to the hippocampus and striatum (Luo et al., 2014). In another study, electroacupuncture (EA) was applied to the acupoints ST36 and LI11 at 1/20 Hz to treat MCAO rats. At 7 and 14 days after the induction of ischemia, the number of BrdU/glial fibrillary acidic protein (GFAP, a marker of astrocyte activation) double-positive cells in SVZ and BrdU/NeuN (a marker of mature neurons) and double-positive cells in the striatum was higher in EA-treated rats (Tao et al., 2010). Liao et al. (2017) also obtained similar results by applying 2 Hz EA in ST36 and ST37 for cerebral infarction rats with ischemia reperfusion injury. These results suggest that EA therapy can induce cell proliferation and differentiation into astrocytes and mature neurons.

Acupuncture mediates the upregulation of neurogenesis-related factors and the activation of related cell signaling pathways that promote the proliferation, migration, and differentiation of NSCs. Tan et al. (2018) found that stimulation at GV20 and *Dazhui* (GV14) by 5/20 Hz EA induced the proliferation and differentiation of endogenous NSCs in MCAO rats, together with upregulating the expression of plasticity-related gene 5 (PRG5, a key factor of neurogenesis) and downregulating the expression of three neurogenesis inhibitors, including the neurite outgrowth inhibitor NogoA, lysophosphatidic acid, and Ras homolog family member A (RhoA), indicating that EA may promote the proliferation and differentiation of endogenous NSCs by regulating PRG5/RhoA signaling. Stimulation with 2 Hz EA at the same acupoints led to the promotion of NSC proliferation and differentiation by brain-derived neurotrophic factor (BDNF), together with vascular endothelial growth factor (VEGF)-mediated angiogenesis of downstream phosphatidylinositol 3-kinase (PI3K) signaling. Specifically, EA increased both proliferation and differentiation in the hippocampus and SVZ and the mRNA and protein levels of BDNF and VEGF in the ipsilateral hippocampus and SVZ, as well as enhancing the levels of phosphorylated PI3K in the newly formed neuroblasts. In addition, the levels of various neurogenesis-related factors were up-regulated, including forkhead box protein G1 (FOXG1), nuclear receptor subfamily 4, group A, member 3 (NR4R3) protein, and zinc finger protein 423 (ZNF423; Wang et al., 2012; Kim et al., 2014). Kim et al. (2018) transplanted bone marrow mesenchymal stem cells (MSCs) into the striata of MCAO mice and stimulated them with 2 Hz EA at GV20 and GV14. The treatment reduced the atrophy of the striatum and promoted the proliferation of NPCs in the SVZ and peristriatal area. Increased numbers of BDNF and neurotrophin-4 (NT-4)-positive neurons were detected in the peristriatal area and the hippocampus. These results indicated that the combined treatment of MSCs and EA could promote neurogenesis in the ischemic brain by upregulating the expression of neurotrophic factors such as BDNF and NT-4, thus improving the motor function of MCAO mice (Kim et al., 2018). Another study in MCAO rats showed that after 1/20 Hz EA at ST36 and LI11 in the affected limb, the number of nestin/GFAP-positive cells was increased in the cortex, as well as the gene and protein levels of Wnt1 and β-catenin, while the transcription of glycogen synthase kinase 3 (GSK3) was inhibited, which suggested that EA may promote the proliferation of nerve stem/progenitor cells in the area around the cortical infarction after stroke through the Wnt/β-catenin pathway, thereby reducing the infarct volume and improving the neurological impairment (Chen et al., 2015). It was also observed that EA significantly enhanced the activity of the neurogenic locus notch homolog protein 1 (Notch1) signaling pathway and its downstream transcription target Hes1, leading to increased NSC proliferation and differentiation (Zhao et al., 2015). Studies have shown that Retinoic Acid (RA) is a key factor in regulating the proliferation of adult hippocampal nerve stem/progenitor cells (Mishra et al., 2018). Hong et al. (2013) found that 5/20 Hz EA at ST36 and LI11 may reduce the infarct volume and promote the recovery of neurological function by regulating the RA pathway in the ischemic brain.

MicroRNAs (miRNAs, miRs) are involved in the post-transcriptional modification of gene expression. In cerebral ischemia/reperfusion injury, acupuncture can promote the regeneration and repair of damaged nerves by regulating miRNAs. Sha et al. found that in rats with cerebral ischemia/reperfusion injury, stimulation with 20 Hz EA at *Waiguan* (TE5) and ST36 on the affected limb increased the expression of miR-223 and reduced the expression of phosphatase and tensin homolog deleted on chromosome ten (PTEN) in the SVZ and hippocampus by activating the Notch1 signaling pathway, thereby increasing the numbers of NSCs (Sha et al., 2019). Another study, *in vivo* and *in vitro*, revealed that EA regulated endogenous neurogenesis through the action of exosomal miRNAs. Specifically, 1/20 Hz EA at ST36 and LI11 on the affected limb induced the differentiation of endogenous NSCs into neurons in the ischemic striatum and SVZ by promoting the release of exosomes containing miR-146b in the ischemic striatum, thereby improving neurological impairment in MCAO rats (Zhang et al., 2020).

Therefore, acupuncture can indeed activate endogenous neurogenesis by stimulating proliferation and differentiation of NSCs in the SVZ and SGZ through activation of related cell-signaling pathways and upregulation of neurogenesis-related factors. It is also worth exploring which acupoints and stimulation parameters are optimal for the induction of neurogenesis. Furthermore, the number of endogenous NSCs is limited and not adequate to generate a sufficient number of differentiated functional neurons, and resolving this obstacle needs further innovative research.

#### Acupuncture promotes axonal regeneration and sprouting

Ischemic stroke is characterized acutely by a massive and rapid loss of axons, and recovery depends on axonal regeneration and sprouting, which constitute the compensatory mechanism to establish the new functional connections and strengthen the communication between neurons (Hinman, 2014). Axonal regeneration involves the regrowth of injured axons that is initiated by the formation of the growth cone at the broken ends, while axonal sprouting is the process of collateral sprouting and elongation of uninjured axons (Darian-Smith, 2009). Therefore, promoting axonal regeneration and the development of sprouting in the right direction contributes to the recovery of ischemic stroke patients.

Growth-associated protein-43 (GAP-43) is a growth cone-associated protein that promotes neurite outgrowth by regulating cytoskeletal organization *via* protein kinase C signaling. The expression of GAP-43 decreases with nerve development, but when the brain is damaged, the neurons adjacent to the injured area compensate for the loss of cells by collateral sprouting and reactive axon regeneration. Under these conditions, the local expression of GAP-43 is again upregulated. Therefore, GAP-43 is considered to represent a molecular marker of axonal growth and plasticity (Felling and Song, 2015). It has been documented that EA at ST36 and *Neiguan* (PC6), performed daily for 20 min, can improve the neurological function in rats with cerebral infarction. The mechanism may be related to the statistically significant upregulation of GAP-43 in the ischemic region, from the 7th day after the injury (Zhou et al., 2011). A similar effect was also obtained by applying acupuncture on both sides of PC6 and GV26 for 7 days (Xu et al., 2017). A study performed in rats showed that bilateral EA at LI11 and ST36 (5 Hz/10 Hz, 2 mA) improved the neurobehavioral score after ischemic stroke more than a unilateral EA, and the expression level of GAP-43 was increased in the CA3 of the hippocampus by EA (Qing et al., 2016). These findings may be explained by the structural organization of the innervation of the limbs. The cerebral cortex innervates the activity of bilateral limbs because 80% of the nerve fibers from the anterior central gyrus innervate the movement of the contralateral limbs, and a small fraction of them, which are not crossed, descend directly to form the anterior corticospinal tract and innervate the movement of the ipsilateral limbs. The secondary fibers of some posterior root fibers are combined with the ascending fibers of the ipsilateral spinothalamic tract, and the bilateral projection effect of the upper and lower fibers of the brain stem network determine the effect of acupuncture signals on the bilateral cerebral cortex. Thus, the cerebral cortex acts as the master center that dominates the movement and sensation of the bilateral limbs (Qing et al., 2016). In addition, another study revealed that 2 Hz EA stimulation of GV20 can not only promote nerve regeneration by upregulating GAP-43 and BDNF after cerebral ischemia but can also reduce inhibition of axon regeneration by downregulation of myelin-associated inhibitors and the RhoA/Rho-associated coiled-coil-containing protein kinase (ROCK) signaling pathway after cerebral ischemia, thus reducing the size of the cerebral infarction and improving neuronal function and hippocampal ultrastructural damage (Chen S. Q. et al., 2020).

MiRNAs are also involved in acupuncture-promoted axonal regeneration. Zhao et al. (2018) have shown that the expression of miR-132 was downregulated after MCAO, and that EA inhibited the transcription of SRY-box transcription factor 2 (SOX2) by upregulating miR-132. These changes resulted in the promotion of axonal sprouting and alleviation of neurological deficits, suggesting that neurobehavioral functional recovery after ischemic injury depends on axonal sprouting enabled by the inhibition of SOX2 translation *via* miR-132 (Zhao et al., 2018). Moreover, it was found that within 28 days after reperfusion in MCAO rats, after 2/10 Hz EA stimulation of GV20, the expressions of paired immunoglobulin-like receptor B (PirB), RhoA, and GAP-43 were regulated by modulating miR-181b directly targeting Pirb mRNA in the penumbra, promoting axon regeneration and new projections of corticospinal tracts after oxygen-glucose deprivation injury, which improved the neurological deficits (Deng et al., 2016).

#### Acupuncture modulates synaptic plasticity

Synapse is not only the specific site of intercellular information transmission but also a sensitive site of neuroplasticity. Synaptic plasticity mostly refers to morphological and functional modifications of synaptic connections, including long-term changes in synaptic structure and number, as well as the short-term changes in the strength and efficiency of neurotransmission (Neves et al., 2008).

##### Plasticity of synaptic structure

The plasticity of the synapsis depends on the modification of its ultrastructure, which includes the following aspects: (1) contact area (total number of synapses, number density of synapses, area density of synapses, average area of single synapse; Mundy et al., 2008); (2) content of active substances, such as synaptophysin (SYN), in synaptic contact area (Kwon and Chapman, 2011); (3) synapse-related subcellular structure, such as the postsynaptic density (PSD)-95 (Xie et al., 2019); and (4) synaptic gap width and interface curvature (Xiao et al., 2018).

Acupuncture has been demonstrated to modify the synaptic structure and promote the synaptic plasticity in rodent models of stroke by upregulating SYN and increasing PSD (Xia et al., 2017; Xiao et al., 2018). After 5 weeks of EA with a 5/10 Hz wave at GV20 and GV14, the thickness of synaptic PSD, and the number and surface densities of synapses were increased in MCAO rats, without significant changes in other indices of synaptic ultrastructure, such as volume density and synaptic curvature. These modifications were accompanied by the upregulation of P38, GAP-43, nerve growth factor (NGF), and BDNF in the ischemic cerebral cortex, indicating that EA could protect synaptic ultrastructure by increasing the level of these proteins (Yi et al., 2006). However, another study found that after 2 weeks of acupuncture in *Taixi* (KI3) and LR3, motor and cognitive deficits were noticeably improved compared with the untreated group at the 7th and 14th day after ischemic stroke. The improvement was accompanied by an increase in BDNF, SYN, PSD, and synaptic curvatures, and a smaller width of the synapse cleft. These results suggested the possibility that the inconsistency in the modifications of the synaptic structure might be caused by differences in acupoints, parameters, target brain areas, and observation points (Xia et al., 2017). Xie et al. (2019) demonstrated that the MCAO injury reduced the number of PSD-95/SYN double-positive cells in the hippocampal CA1 area, and 14 days of EA stimulation of GV20 and GV24 points reversed this trend, thus affecting synaptic plasticity. Activation of the Janus-activated kinase 2 (JAK2)/signal transducer and activator of transcription 3 (STAT3) signaling has been proved to induce spatial learning and memory impairment by inhibiting synaptic plasticity in the hippocampal CA1 region. EA treatment decreased the levels of phosphorylation of JAK2 and STAT3 in the CA1, indicating that the intervention at GV20 and GV24 improved synaptic plasticity by inhibiting the JAK2/STAT3 signaling pathway (Xie et al., 2019). In addition, after 1/20 Hz EA at the same acupoints, it was found that the density of dendritic spines was increased and the cognitive function of MCAO rats was improved. It was suggested that the mechanism may be related to the upregulation of Cdc42, RAS-related C3 botulinus toxin substrate 1 (Rac1), and Filamentous actin (F-actin) in the hippocampus, as well as down-regulation of RhoA expression (Lin et al., 2016).

##### Plasticity of synaptic function

It has been proposed that the plasticity of synaptic function is dependent on its structural plasticity. Functional plasticity is mostly related to the strength of synaptic transmission efficiency in the form of long-term potentiation (LTP) and long-term depression (LTD), which are prevalent paradigms of micro-neuroplasticity (Matsuzaki et al., 2004). LTP facilitates synaptic transmission, amplifies presynaptic signals, and enhances the persistence of postsynaptic potentials, thereby appearing more relevant to synaptic remodeling than LTD (Kim E. et al., 2012; Stewart and Dringenberg, 2016). Cognitive impairment often occurs after ischemic stroke and leads to difficulties in memory, learning, analysis, organization, interpretation, and concentration, decreasing the quality of life (Liu F. et al., 2014), which is the main cause of persistent sequelae of ischemic stroke. In these processes, LTP can enhance long-term memory through acquisition, consolidation, and storage of information, while LTD can verify the memory content and regulate LTP (Neves et al., 2008; Xiao et al., 2018).

Dendritic spines contribute to the spread of information *via* deformation and control synaptic efficacy by modulating LTP and LTD that are related to learning and memory (Schacher and Hu, 2014; Park et al., 2015). It has been reported that LIM domain kinase 1 (LIMK1) regulates LTP and long-term memory by activating cyclic adenylate monophosphate response element-binding protein (CREB), and LIMK1 knockout mice suffer from the severe damage of dendritic spines and LTP in the hippocampus. MiRNAs, which play important roles in learning and memory formation, can regulate LIMK1 expression to induce synaptic-dendritic plasticity. Therefore, the modulation of miRNA-LIMK1 represents a potential target for the treatment of cognitive deficit. Liu et al. (2017) demonstrated by T2-weighted imaging that EA at GV20 and GV24 alleviated cognitive impairment and reduced the volume of multiple brain lesions. Importantly, synaptic-dendritic loss in hippocampal CA1 pyramidal cells could be rescued by EA. This effect was associated with the suppression of the increase in the expression of miR-134 and LIMK1, indicating that these molecules might mediate the induction of hippocampal synaptic plasticity by EA, which contributed to the improvement of learning and memory during the recovery from ischemic stroke (Liu et al., 2017). In addition, acupuncture could lead to lasting changes in cortical excitability by affecting the activity of neurosynapses (Yang et al., 2017). For instance, a study in which 18 healthy subjects were subjected to acupuncture at the LI11 and TB5 acupoints showed an increased muscle excitability that lasted for 20 min after the needle was pulled out, proving the existence of a time-dependent effect of acupuncture on the excitability of bilateral primary motor cortex, which can induce LTP-like plasticity and increase motor learning (He et al., 2019).

Numerous studies have demonstrated that acupuncture enhances LTP in rats subjected to MCAO. For example, EA at GV20 for 6 days can reverse the MCAO-induced impairment of memory and decrease in hippocampal LTP, and inhibit the overexpression of N-methyl-d-aspartate receptor subtype 1 (NR1) and transient receptor potential vanilloid subtype 1 (TRPV1) receptors in the CA1 area of the hippocampus, indicating that NR1 and TRPV1 may mediate the effect of EA on MCAO-induced behavioral defects and LTP damage (Lin and Hsieh, 2010). Ye et al. (2017) have documented that acupuncture at GV20 and ST36 restored cognitive dysfunction in the rat two-vessel occlusion (2VO) model. The primary mechanism underlying this acupuncture effect was the improvement of decreased LTP by promoting the release of dopamine and its main metabolites and reversing the decline in dopamine D1/D5 receptors (Ye et al., 2017).

This section summarized the evidence that acupuncture regulates structural plasticity after stroke, that is, acupuncture can stimulate neurogenesis, axonal regeneration and sprouting, improve the structure and function of the synapse, to provide a theoretical basis for the improvement of the neurological deficits of ischemic stroke by acupuncture.

### Glial Cells Play A Key Role in The Regulation of Neuroplasticity by Acupuncture

Glial cells, including astrocytes, oligodendrocytes, and microglia, play a key role in acupuncture’s regulation of neuroplasticity. By interacting with neurons, they regulate the formation and remodeling of synapses and neural circuits, thus contributing to the reconstruction of the functional brain (Stogsdill and Eroglu, 2017). Acupuncture can regulate neuroplasticity through glial cells, and the evidence is shown in Table 2.

**Table 2:** The mechanisms of glial cells in the regulation of neuroplasticity by acupuncture in the treatment of ischemic stroke.

**Study**	**Model/Objects**	**Intervention**	**Acupoints**	**Comparison**	**Acupuncture parameters**	**Effect measurements**	**Biochemical measurements**
Kim et al. (2012)	MCAO rats	EA	GV20, GB7	Non-EA	3 Hz, 5 min, 2/7/14 days	Garcia scale scores↑	BDNF↑
Cheng et al. (2014)	MCAO rats	EA	GV20, GV14	Non-EA	5 Hz, 2.7–3.0 mA, 25 min, 6 days from 24 h after reperfusion	Cerebral infarct area↓; neurological deficit scores↓	Penumbra area: GFAP^+^ cells↓, S100B^+^ cells↓, NF-κB (p50)^+^ cells↓, TNF-α^+^ cells↓, iNOS^+^ cells↓, nuclear NF-κB (p50)↓, GFAP/S100B immunoreactivity↓, S100B/nitrotyrosine immunoreactivity↓, TUNEL^+^ cells↓, cytosolic GFAP↓, p-p38 MAPK↓, cytosolic TRADD↓, FADD↓, cleaved caspase-8↓, cleaved caspase-3↓
Zhao et al. (2013)	MCAO rats	EA	BL23, BL17, GV20	Non-EA	1/7 days	-	Hippocampus: BDNF mRNA↑
Yi et al. (2006)	MCAO rats	EA1: 2 weeks after ischemia, EA2: 5 weeks after ischemia	GV14, GV20	Non-EA	5-10 Hz, 30 min, 2/5 weeks from MCAO immediately	-	Synaptic numerical density (EA2)↑, synaptic surface density↑, PSD(EA2)↑, synaptic cleft width↑, P38(EA2)↑, NGF↑, BDNF↑
Tao et al. (2016)	MCAO rats	EA	LI11, ST36	Non-EA	1/20 Hz, 30 min, 3 days from 24 h after MCAO	Neurological deficit scores↓; cerebral infarct volumes↓; average speed of catwalk↑; mean duration on the rotarod↓	Peri-infarct cortex and ST: GFAP^+^ /Vimentin^+^ cells↑, GFAP^+^ /Nestin^+^ cells↑, GFAP^+^ /BrdU^+^ cells↑, Cyclin D1↑, CDK4↑, p-Rb↑, BDNF↑
Huang et al. (2017)	MCAO rats	EA	GV20, GV24	Non-EA	2/20 Hz, 0.2 mA, 30 min, 7 days from the 12 h after MCAO	Neurological deficit scores↓; mNSS↓; escape latency↓; swimming speed↓; target crossing↑; cerebral infarct volumes↓	Peri-infarct hippocampal CA1 and sensorimotor cortex: ED1^+^ cells↓, GFAP^+^ cells↓, IL-1β↓, IL-10↑, P2X7R^+^ /ED1^+^ cells↓, P2X7^+^ /GFAP^+^ cells↓, P2Y1R^+^ /ED1^+^ cells↓, P2Y1R^+^ /GFAP^+^ cells↓

Notes: ↑, upregulated by acupuncture; ↓, downregulated by acupuncture. Abbreviation: GB20, Fengchi; GB39, Xuanzhong; LI11, Quchi; LI4, Hegu; ST36, Zusanli; SP6, Sanyinjiao; GV20, Baihui; FMA, Fugl-Meyer Assessment; GV24, Shenting; MICD, middle cerebral artery occlusion induced cognitive deficit; MWM test, Morris water maze test; GB33, Xiyangguan; ReHo, regional homogeneity; BA, Brodmann Area; NDS, nervous functional deficiency scale; MBI, modified Barthel index; MCAO, middle cerebral artery occlusion; CPu, Caudate putamen; MCTX, motor cortex; SCTX, somatosensory cortex; FC, functional connectivity; LPs, superior parietal lobule; SMA, Supplementary Motor Area; TE5, Waiguan; GB7, Qubin; M1, Primary motor area; PMC, premotor cortex; RSC, retrosplenial cortex; GMV, Grey Matter Volume; ISSA, International Standard Scalp Acupuncture; PG, parahippocampal gyrus.

#### Glial cells regulate neuroplasticity by secreting NTFs

It is well documented that NTFs are required for neurogenesis, axonal regeneration, synaptic activity and plasticity, and other neuroplasticity processes in CNS diseases, and can improve neurological deficits. NGF, BDNF, neurotrophin-3, and NT-4 are four members of the mammalian neurotrophin family (Gibon and Barker, 2017).

##### BDNF

The role of BDNF in neuroplasticity makes it an excellent candidate for stroke treatment (Mang et al., 2013). Additionally, BDNF acts on NSCs, promoting their survival, proliferation, and differentiation by interacting with TrkB and initiating downstream pathways, such as mitogen-activated protein kinase (MAPK)/extracellular signal-regulated kinase (ERK) and PI3K/Akt cascades. MAPK/ERK and PI3K/Akt signaling can reduce neuronal death induced by excitotoxicity in the ischemic penumbra (Tejeda et al., 2019). BDNF can also increase the formation of dendrite branches and connections between dendrites and promote the development and maturation of the CNS (Song et al., 2017). BDNF is directly involved in angiotensin-1 receptor blocker-mediated functional recovery, angiogenesis, and synaptic formation (Fouda et al., 2017). Finally, BDNF can provide local anti-inflammatory effect by downregulating the expression of tumor necrosis factor alpha (TNF-α) and upregulating the expression of interleukin-10 (IL-10), to promote nerve repair. BDNF increases the number of activated and phagocytic microglia *via* the increase in intracellular Ca^2+^. Once activated, microglia can further secrete BDNF and other NTFs, generating a positive feedback loop (Jiang et al., 2010).

In animal models of MCAO, acupuncture utilizing different parameters, such as acupoints, frequency, and intervention time, produces distinct effects on BDNF in the brain. The EA treatment at the GV20 and *Qubin* (GB7) acupoints for 14 days after MCAO, utilizing a bipolar waveform current of 3 Hz applied for bursts of 5 s and 2 s intervals, increased the levels of BDNF in the ischemic lobe and improved functional and motor recovery (Kim M. W. et al., 2012). Similarly, EA at GV20 and GV14 for two times with 5 Hz/10 Hz enhanced the expression of BDNF in the ischemic area (Cheng et al., 2014; Chavez et al., 2017). In MCAO mice, the expression of BDNF mRNA in the hippocampus was upregulated by EA applied for seven consecutive days at the *Shenshu* (BL23), *Geshu* (BL17), and GV20 acupoints (Zhao et al., 2013). Furthermore, EA at the GV20 once a day for 2 weeks improved motor function, elevated BDNF in the ischemic hemisphere, and increased the number of BDNF-positive cells and the expression of TrkB (Kim M. W. et al., 2012). Thus, acupuncture may play a neuroprotective role in ischemic stroke by modulating the expression of BDNF, but the functional verification of this possibility remains to be obtained.

##### NGF

NGF is the first NTF identified by Rita Levi-Montalcini and Viktor Hamburger in mouse sarcoma *in vitro* in the 1950s. Subsequent studies have established that NGF binds to the tropomyosin-related kinase A (TrkA) and p75NTR receptors to activate downstream signaling cascades, such as MAPK/ERK and PI3K/Akt. The stimulation of these pathways modulates the transcription of neurite outgrowth inhibition protein A, Nogo receptor, Rho family small GTPases A, Rho-associated kinase 2, thus promoting the differentiation and survival of neurons and growth of axon growths (Chang et al., 2016; Keefe et al., 2017). Furthermore, NGF exhibits neuroprotective activity by reducing intracellular calcium overload and antagonizing the excitotoxicity of amino acids after cerebral ischemia (Keefe et al., 2017).

Yi et al. (2006) demonstrated that EA at the GV20 and GV14 points increased the expression of NGF after ischemic brain injury. A higher level of NGF was essential to protect neurons by promoting synaptic plasticity and axonal regeneration, reflected by the increase of numerical density, surface density, volume density, and gap width of cortical synapses, as well as PSD and GAP-43 expression in the ischemic area. The results also documented an increase in expression of NGF from 2 to 5 weeks after EA intervention, indicating that acupuncture can not only increase NGF but also maintain its expression at a high level for a prolonged time, achieving a long-lasting protective effect (Yi et al., 2006).

Other NTFs, such as glial cell line-derived neurotrophic factor (GDNF; Manni et al., 2011), VEGF, insulin-like growth factor (IGF; Liu S. J. et al., 2014), stromal cell-derived factor-1a, and ciliary neurotrophic factor (Kim et al., 2013), have also been demonstrated to mediate the regulatory effects of acupuncture on neuroplasticity. Therefore, the ability of acupuncture to enhance neuroplasticity and improve neural function defects appears to be dependent on the NTFs family of growth factors (Manni et al., 2010; Xiao et al., 2018). In addition to the secretion of neurotrophic factors, other mechanisms by which astrocytes and microglia influence neuroplasticity are also worthy of attention.

### Mechanisms of astrocyte regulation of neuroplasticity

Astrocytes have an important effect on neuroplasticity. In addition to secreting NTFs, astrocytes can also secrete or express specific mediators that regulate the number, morphology, and function of synapses, such as Maverick (a member of TGF-β superfamily), Hevin/Sparcl1, and thrombospondin. Cholesterol synthesized by astrocytes can significantly enhance the transmission efficiency of synapses, which is also promoted by glutamic acid and homocysteine released by astrocytes. D-serine plays an essential role in the induction of LTP without apparent effects on normal synaptic transmission (Allen and Eroglu, 2017). On the other hand, reactive astrocytes subsets residing within hundreds of microns from the infarcted area are involved in the formation of glial scar separating the infarct from the surrounding intact brain tissue. However, the overgrowth of astrocytes is detrimental because the upregulation of extracellular matrix proteins and other inhibitors of cell growth can prevent the effective regeneration of axons (Silver and Miller, 2004). For example, the deposition of chondroitin sulfate proteoglycans released by astrocytes *via* RhoA/ROCK-mediated pathways restricts neuroplasticity in the proximity of the glial scar, permitting the reconstruction of the neural circuit only distal sites (Carmichael et al., 2005; Sims and Yew, 2017).

The number of cells double-positive for GFAP/BrdU, GFAP/vimentin (expressed in radial neuroglia cells and neural and glial precursors), and GFAP/nestin in the cortex and striatum of MCAO rats was further increased 3 days after the EA of LI11 and ST36, indicating that EA can promote the proliferation of astrocytes in the cortex and striatum. At the same time, increased expression of cyclins, including cyclin Dl, cyclin-dependent kinase 4 (CDK4), and phospho-Rb, in the cortex and striatum regulates the activation of the G1-to-S transition, suggesting reactive astrocyte proliferation. In addition, EA enhanced the local expression of BDNF in the cortex and striatum. Together, these results indicated that EA plays a neuroprotective role by stimulating the proliferation of GFAP/vimentin/nestin-positive reactive astrocytes, which, in turn, secrete BDNF (Tao et al., 2016). It may be reflected in improving the structural characteristics of the synaptic interface, including numerical density, surface density, volume density, and gap width of the cortical synapse, enhancing the transmission function of the synapse, and modifying its structure and function, thus stimulating the regeneration of nerves and repair of the injury (Allen and Eroglu, 2017). At a later stage, acupuncture can increase the formation of new neurons and inhibit the excessive proliferation and differentiation of astrocytes, preventing the inhibition of axon regeneration by the excess of harmful substances (Jiang et al., 2016). It has been demonstrated that in the traumatic brain injury model not in cerebral infarction model, the number of BrdU/GFAP double-positive cells in the rats after acupuncture at GV20, GV26, *Fengfu* (GV16), GV15, and bilateral LI4 acupoints was significantly higher than that in the untreated group, which decreased to the same level as that in the untreated group at 7 days, reaching the same value as that of the sham-operated rats at 14 days, while the level remained higher in the injured animals not treated with acupuncture. These investigations demonstrated that acupuncture is conducive to the moderate proliferation and differentiation of astrocytes.

### Mechanisms of microglia regulating neuroplasticity

Resident microglia perform a variety of functions in response to the pathological changes associated with the ischemic injury. They can rapidly change their morphology and migrate to the site of injury, affecting the integrity of neurons, changing synaptic input and activity, and activating neurogenesis. All these processes are beneficial to the recovery and reconstruction of neural function (Pocock and Kettenmann, 2007; Morrison and Filosa, 2013). Activated microglia complement receptor 3 can trigger LTD of peripheral neurons by regulating nicotinamide adenine dinucleotide phosphate oxidase, one of the main mediators of stroke neurotoxicity (Zhang et al., 2014). Another study showed that after cerebral ischemia, the activity-dependent connection between microglia and synapses was significantly prolonged for approximately 1 h, while the connection in the intact brain was maintained for approximately 5 min, suggesting that microglia can regulate LTP. This ability of microglia is critical for the reconstruction of the neural circuit after cerebral ischemic injury (Wake et al., 2009). Stimulation of microglia with low levels of interferon-gamma (IFN-γ) promotes early neurogenesis after stroke (Butovsky et al., 2006). In the chronic stage of ischemia, 16 weeks, the expression of IGF-1, GDNF, and BDNF in the M2 microglia in the ipsilateral SVZ (Ekdahl et al., 2009), generates conditions conducive to the continuous neurogenesis and recovery of function (Neumann et al., 2006; Nakajima et al., 2008). Additionally, microglia secrete chemokines, such as stromal cell-derived factor-1 α, which can bind to its receptor C-X-C chemokine receptor 4 (CXCR4) that is highly expressed in neural progenitor cells, promoting the migration of NSCs to the infarcted area.

At present, most of the research on the action of acupuncture on the neurological deficits of ischemic stroke has been from the perspective of reducing neuroinflammation, represented by reducing microglial activation and the release of proinflammatory mediators and neurotoxic substances. For example, Huang et al. (2017) performed EA on GV20 and GV24 for 7 days, and the results showed that EA could improve the neurological deficits and motor function of MCAO rats, as well as playing an anti-inflammatory role by reducing the secretion of interleukin-1beta (IL-1β) and promoting the release of IL-10 (Huang et al., 2017). However, there is little clinical and experimental evidence of the role of microglia in the acupuncture-mediated modulation of neuroplasticity after ischemic stroke, and this should be addressed in future research.

In conclusion, the role of glial cells in neuroplasticity has become an important research topic. These cells not only secrete NTFs and other factors but also transmit signals through their interaction with neurons. However, much is still not understood, specifically, the involvement of glial-neuronal interactions on the regulation of neuroplasticity by acupuncture.

## Acupuncture Promotes The Functional Reorganization of Different Brain Regions

The functional reorganization of different brain regions after cerebral ischemic injury depends on the plasticity of both individual neurons and the functional networks of the brain, including contralateral transfer, ipsilateral functional compensation, and the activation of latent pathways. Experimental models of stroke in mice, rats, and monkeys allowed the identification of new connections formed in the ipsilateral cerebral hemisphere between the motor, somatosensory, and premotor areas of the cerebral cortex adjacent to the stroke. Stroke can also induce new connections between frontal motor regions and parts of the brainstem or spinal cord that have lost their projections from the stroke region in the contralateral hemisphere (Carmichael et al., 2017). At the same time, increased cerebral blood flow and excitability in the contralateral cortex contribute to recovery from stroke. There is simultaneous compensation within the ipsilateral and contralateral hemispheres, although this is dependent on the dominant hemisphere. For example, when a cortical lesion is large, the compensatory effect of the ipsilateral residual cortex is weakened, while the contralateral compensatory effect is enhanced (Fang et al., 2012). The functional compensation of both the tissue surrounding the lesion and the contralateral hemisphere may be explained by the activation of latent pathways or dormant synapses (Xiao et al., 2017). Acupuncture has been suggested to be complementary to mainstream rehabilitation after stroke, promoting the improvement of motor, sensory, cognitive, and other impairments, and is closely related to the activation of the corresponding brain areas (Li et al., 2015; Wu J. et al., 2017; Wen et al., 2018). This section explains the underlying functional plasticity mechanism activated by acupuncture during the regulation of neurological deficits after stroke, as shown in Table 3.

**Table 3:** The mechanisms of acupuncture on regulating functional reorganization in the treatment of ischemic stroke.

**Study**	**Model/Objects**	**Intervention**	**Acupoints**	**Comparison**	**Acupuncture parameters**	**Effect measurements**	**Biochemical measurements**
Li et al. (2015)	Stroke patients with unimanual motor deficits (*n* = 14)	Acupuncture + conventional treatment	GB20, GB39, LI11, LI4, ST36, SP6, (bilateral), GV20	Conventional treatment	30 min, 5 days, 4 weeks	FMA↑	Diffusion indices values were changed in the corpus callosum and bilateral corticospinal tracts, the inferior longitudinal fasciculus, the inferior frontooccipital fasciculus, the superior longitudinal fasciculus, the forceps minor, the cingulum gyrus, and the thalamic radiation.
Wen et al. (2018)	MICD rats	EA	GV20, GV24	Non-EA	1/20 Hz, 6 V, 2 mA, 30 min, 14 days from 24 h from MICD	MWM test: the time to find the platform↓, the number of times the platform crossed↑	The activation of hippocampus, retrosplenial cortex, cingulate gyrus, prelimbic cortex, and sensory cortex↑
Wu et al. (2017)	Ischemic stroke patients (*n* = 21)	Acupuncture + conventional treatment	GV20, GB20, LI11, LI4, ST36, GB33, GB39, SP6	Conventional treatment	30 min, 5 days, 4 weeks	NDS↓, FMA↑, MBI↑	ReHo in the frontal lobe (BA6, BA46), supra-marginal gyrus (BA40), middle temporal gyrus (BA21), cerebellum, and insula↑
Wu et al. (2018)	MCAO rats	EA	LI11, ST36	Non-EA	2/20 Hz, 30 min, 7 days from 24 h after reperfusion	Neurological deficits↓; the infarct volumes↓; catwalk gait and rotarod test: the motor function↑	The neural activity of CPu, MCTX, SCTX regions↑
Liang et al. (2017)	MCAO rats	EA	ST36, LI11	Non-EA	1/20 Hz, 6 V, 2 mA, 30 min, 7 days from 24 h after MCAO	Neurologic deficits↓; the cerebral infarctions↓	The neural activity of motor cortex, dorsal thalamus, and ST↑
Li et al. (2021)	MCAO rats	EA	ST36, LI11	Non-EA	2/20 Hz, 30 min, 14 days from 24 h after MCAO	Infarct volumes↓; mNSS↑; motor functional performances↑	The FC between the left motor cortex and the motor function-related brain regions, including the motor cortex, sensory cortex and ST↑
Chen et al. (2020)	Patients with ischemic stroke in the left hemiplegia (*n* = 10)	MA	ST36, LI11	Self-control	Twisting for ± 180°, 60 circles per min, 15 min	-	ReHo values at the right precentral gyrus and superior frontal gyrus↑, ReHo values at right LPs, left fusiform gyrus and left SMA↓
Chen et al. (2014)	Patients with ischemic stroke in the left basal ganglia (*n* = 24)	Acupuncture	TE5	Sham acupuncture, non-acupoint acupuncture	Twisted for ± 180°, 60 circles, 6 min and 30 s	-	The sensorimotor network of the ipsilesional hemisphere is regulated, the contralesional sensorimotor network is stimulated, the cooperation of bilateral sensorimotor networks is increased, and the synchronization between the cerebellum and cerebrum is changed.
Huang et al. (2013)	Patients with ischemic stroke in the left hemisphere (*n* = 10)	MA	Right TE5	Tactile stimulation	30-s twist and 30-s rest, six times, 60 circles per min	-	The activation of the postcentral gyrus (BA2, BA3), precentral gyrus (BA4, BA6), medial frontal gyrus (BA6)↑, the postcentral (BA3), precentral gyrus (BA6)↓
Huang et al. (2012)	Patients with ischemic stroke in the left internal capsule (*n* = 43)	MA	Right TE5	Sham needling, Non-needling	Twirling the needling 180°, 60 times, 3 min	-	The activation of BA30↑ (Compared with the non-needling group), BA13, 19, and 47↑ (Compared with sham needling at TE5), BA9↓ (Compared with needling at the sham point)
Fang et al. (2012)	Patients with ischemic stroke in the right basal ganglion region (*n* = 6)	EA	GV20, right GB7	Self-control	2 Hz, 20 min	-	First EA treatment: the activation of M1, PMC, LPs bilaterally, SMA↑ on the unaffected right hemisphere 3 weeks of EA treatments: Bilateral M1 and LPs significantly↑, insula, putamen, and cerebellum↑
Zhang et al. (2021)	MICD rats	EA	GV20, GV24	Non-EA	1/20 Hz, 6 V, 0.2 mA, 30 min, 14 days from 24 h after MICD	MWM test: escape latency times↓, the number of times the platform crossed↑	FC between the RSC and hippocampus, cingulate gyrus and midbrain↑
Wu et al. (2018)	Ischemic stroke patients	Acupuncture + conventional treatment	GV20, GB20, LI11, LI4, GB33, ST36, SP6, GB39	Conventional treatment	30 min, 5 days/week, 4 weeks	MBI scores↑	The GMV of left frontal lobe, precentral gyrus, superior parietal gyrus, anterior cingulate cortex, middle temporal gyrus↑, the right frontal gyrus, inferior parietal gyrus, middle cingulate cortex ↓
Chen et al. (2013)	Patients with ischemic stroke in the left hemisphere (*n* = 10) and six healthy controls (*n* = 6)	EA	Right TE5	Self-control	Twirling the needle ± 180°, 60 times, 6 min	-	The activation of left BA5, 6, 7, 18, 19, 24, 32, hypothalamus, the ventral posterolateral nucleus, the right BA4, 6, 7, 18, 19, 32↑, left BA13, the hypothalamus, posterior lobe of the tonsil of cerebellum, the culmen of the anterior lobe of hypophysis, right BA13↓; the deactivation of the left BA6, 11, 20, 22, 37, 47, the culmen of the anterior lobe of hypophysis, alae lingulae cerebella, the posterior lobe of the tonsil of cerebellum, right BA8, 37, 45, 47, the culmen of the anterior lobe of hypophysis, nodule of the anterior lobe of hypophysis, inferior border of the lentiform nucleus, lateral globus pallidus, the parahippocampal gyrus↑, BA7↓
Liu et al. (2020)	Patients with acute infarction in the middle cerebral artery supply area in the dominant hemisphere (*n* = 30)	MA + conventional treatment	ISSA, needling at the parietal midline (MS5) and left anterior/posterior parietal-temporal oblique lines (MS6 and MS7)	Conventional treatment	Twisting at 200 turns, 30 min, two times a day, six days	NIHSS scores↑; motor function scores↑	Centered to the seed region of the left SMA, FC at the left middle cerebellar peduncle, left cerebellum posterior lobe (uvula and declive), vermis, fusiform gyrus, lingual gyrus, inferior occipital gyrus, calcarine, cuneus, precuneus, BA7, BA18, and BA19, etc↑; Centered to the seed region of the left PG, FC at the left precuneus, inside-paracingulate, inferior parietal gyrus, paracentral lobule, BA5, BA6, BA7, and BA40, right median cingulate, precuneus, BA19, BA23, and BA31, etc↑.

Notes: ↑, upregulated by acupuncture; ↓, downregulated by acupuncture. Abbreviation: MCAO, middle cerebral artery occlusion; EA, electroacupuncture; GV20, Baihui; GB7, Qubin; BDNF, brain-derived neurotrophic factor; GV14, Dazhui; GFAP, glial fibrillary acidic protein; NF-κB, Nuclear Factor kappa B; TNF-α, Tumour Necrosis Factor alpha; iNOS, Inducible nitric oxide synthase; MAPK, mitogen-activated protein kinases; TRADD, TNFR1-associated death domain protein; FADD, Fas-associated protein with death domain; BL23, Shenshu; BL17, Geshu; PSD, post-synaptic density; NGF, Nerve growth factor; LI11, Quchi; ST36, Zusanli; BrdU, 5-bromo-2’-dexoyuridine; CDK4, cyclin-dependent kinase 4; p-Rb, phosphorylated retinoblastoma protein; IL-1β, interleukin-1beta; IL-10, interleukin-10; P2X7R, P2X7 receptor; P2Y1R, P2Y1 receptor.

Numerous imaging studies have revealed that acupuncture can induce changes in bilateral brain regions or functional connections between brain regions by stimulating unilateral limbs, thus improving sensorimotor disorders after ischemic stroke. Animal experiments showed that after EA treatment at the LI11 and ST36 acupoints of the affected limbs, the infarct volume of MCAO rats was significantly reduced and motor function was recovered. It has been suggested that the possible reason for this is that neural activity and functional connections among brain regions related to motor function were stimulated by EA, including the caudate putamen, motor cortex, somatosensory cortex, dorsal thalamus, and striatum (Liang et al., 2017; Wu P. et al., 2017; Li et al., 2021). Using regional homogeneity (ReHo) analysis in a resting state in patients, it was found that the ReHo values of the right precentral gyrus and superior frontal gyrus were increased, and the ReHo values of the right superior parietal lobule, left fusiform gyrus, and left supplementary motor area were decreased after needling LI11 and ST36 of the right (healthy) limb, indicating that needling one side could stimulate changes in neuronal activity in bilateral brain regions (Chen S. et al., 2020). Acupuncture at the TE5 acupoint can regulate the sensorimotor network of the ipsilateral hemisphere, stimulate the contralateral sensorimotor network, enhance the synergy of the bilateral sensorimotor network, and alter the synchronicity between the cerebellum and the brain (Chen L. et al., 2014). The application of acupuncture together with twisting manipulation affected broad areas of the brain, influencing the contralateral intact hemisphere in patients with unilateral cerebral apoplexy of the primary sensorimotor area and the medial frontal cortex, which suggested that the effects of acupuncture are redistributed throughout the cortex, including the unaffected hemisphere, thereby enhancing the effect of the compensatory process (Huang et al., 2013). For example, the brain region responsible for motor execution and the limbic system of the affected hemisphere were activated by acupuncture at TE5, as well as the brain region related to motor execution and the cerebellum of the unaffected hemisphere. At the same time, improving glucose metabolism in the affected hemisphere also contributes to brain compensation (Huang et al., 2012). EA at GV20 and right GB7 resulted in immediate changes in glucose metabolism in both hemispheres in response to excitatory changes in various brain regions, including bilateral effects in the primary motor area (M1), premotor cortex, and superior parietal lobule (LPs), as well as the supplementary motor area in the unaffected hemisphere. The effect was still apparent 3 weeks after the acupuncture treatment with significant changes observed in glucose metabolism in bilateral M1 and LPs, as well as in other sites such as the insula, putamen, and cerebellum (Fang et al., 2012). These studies show that acupuncture activated bilateral motor areas of the brain, as well as neural tissues related to motor activity, to promote motor function after ischemic stroke. There is, thus, functional reorganization of multiple affected and unaffected areas of the cerebral cortex, and activation of potential functional areas to enhance compensation, especially in the unaffected hemisphere.

EA at GV20 and GV24 can improve cognitive dysfunction in rats after ischemic stroke, and its protective effect may be related to the activation of cognition-related brain regions, such as the hippocampus, retrosplenial cortex, cingulate gyrus, prelimbic cortex, and sensory cortex (Wen et al., 2018), as well as increasing the functional connectivity of these regions (Zhang et al., 2021). Other studies have reported that acupuncture can cause the structural restructuring of the network of the frontal lobe and default mode network (Wu et al., 2018). These brain regions may be the potential therapeutic targets and mechanisms for acupuncture to promote motor and cognitive recovery. In addition to movement, sensation, and cognition, acupuncture can also regulate brain regions related to visual function. For example, acupuncture at TE5 resulted in modulation of brain regions such as BA18 and BA19 (Chen et al., 2013). The International Standard Scalp Acupuncture, namely, acupuncture of the parietal midline (MS5) and left anterior/posterior parietal-temporal oblique lines (MS6 and MS7), can regulate functional brain connections in the dominant hemisphere of patients with acute middle cerebral artery infarction, and specifically strengthen the connections among vision-related brain regions such as BA18 and BA19, and the calcarine, fusiform, and lingual gyri (Liu et al., 2020). At present, most of the imaging studies on the use of acupuncture in the treatment of ischemic stroke have focused on improving the relationships between motor, sensory, and cognitive impairment, and functional compensation in bilateral brain regions, while the neurophysiological mechanism underlying the functional integration of the various CNS components after ischemic stroke remains to be investigated.

## Discussion and Conclusion

The selection of acupoints is critical to the acupuncture process. Clinical investigations have emphasized specific treatments and have aimed to verify the effectiveness of acupuncture for the different sequelae of ischemic stroke, such as hemiplegia, dysphagia, and cognitive impairment. Although acupuncture treatments may vary according to the different types of functional impairment, they all comply with the principle of integral-local combination, that is, the acupoints in the functional areas of the brain are matched with those in specific lesion areas. These acupoints are located on yang meridians, especially Yangming and Shaoyang meridians, which have been verified by Dr. Zhang et al. (2018) through data mining technology (Zhang et al., 2018). Basic research solves a specific scientific question and emphasizes standardized acupuncture techniques and repeatability, resulting in differences between the treatment protocols in clinical and basic research. The selection of acupoints in basic research partially follows the clinical principle of integral-local combination, and many studies use head or body acupoints alone. Single or no more than five acupoints are generally used in basic research, with most of the selected acupoints used being acupoints that have been found to be effective in clinical practice, such as GV24, GV20, ST36, LI11, and TE5.

The neuroprotective effect was influenced by the different frequencies and waveforms of EA. Through the analysis of the data, we found that density-sparse waves were the most used, such as 1/20 Hz and 2/10 Hz. Previous studies have shown that the neuroprotective effect generated by density-sparse waves is the most marked, followed by intermittent waves, with continuous waves performing the worst. The reason may be that the density-sparse wave can activate different signaling pathways through conversion between low-, medium-, and high-frequency stimuli, to stimulate the release of different types of neurochemicals, leading to the neuroprotective effect, while the continuous wave can induce tolerance to electrical stimulation (Li and Wang, 2013). In addition, the acupuncture period is also one of the important factors affecting the curative effect of acupuncture. We observed that EA for 5 and 30 min has a superior effect in reducing the sizes of the ischemic cerebral infarction and neurological deficits, consistent with the conclusions of previous studies (Zhou et al., 2013).

The intervention time window of acupuncture is of great significance to the prognosis of ischemic stroke. In this review, most of the acupuncture intervention time point in the included basic studies was 24 h following the induction of MCAO, but a few studies carried out acupuncture intervention at 3 and 5 days after modeling. The duration of acupuncture ranged from 3 to 21 days. In terms of clinical research, a systematic review and network meta-analysis on the efficacy and safety of dissimilar acupuncture intervention time-points in treating stroke found that the faster the acupuncture intervention, the more effective it is in improving FMA score and BI. The optimal time point for acupuncture intervention is within 48 h after stroke, and the significant validity period lasts until 15 days after onset (Zhuo et al., 2021). Therefore, both clinical and basic studies suggest that acupuncture treatment within 24 and 48 h after cerebral infarction is more beneficial. The period of acupuncture treatment was less than 3 weeks due to greater self-recovery ability in animal models of cerebral infarction. Comparatively speaking, the clinical stroke patients have a longer course of disease, so in addition to giving acupuncture as early as possible, longer acupuncture period can be implemented to promote neuroplasticity and improve the sequelae of ischemic stroke.

**Figure 2:**
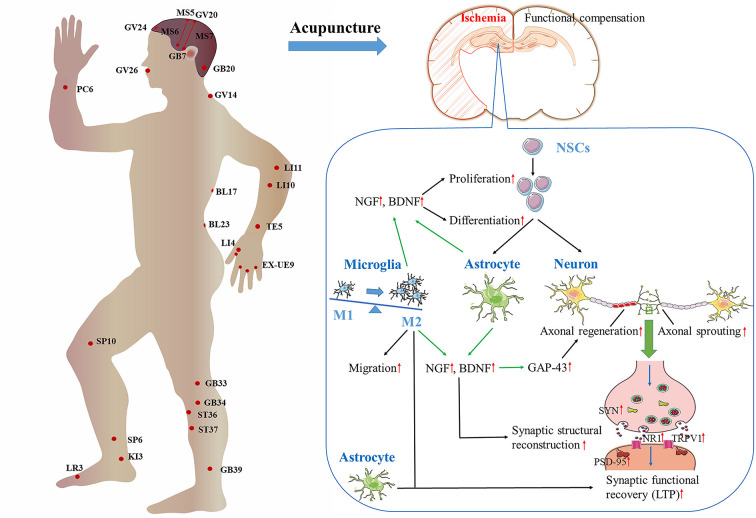
Schematic representation showing the modulation of neuroplasticity by acupuncture after ischemic stroke by the promotion of functional compensation and structural plasticity. The data of acupoint selection and mechanism are derived from the mechanistic studies by using human and rodents. PC6, *Neiguan*; GV26, *Shuigo*u; GV24, *Shentin*g; GV20, *Baihui*; GB7, *Qubin*; GB20, *Fengchi*; GV14, *Dazhui*; LI11, *Quch*i; LI10, *Shousanli*; TE5, *Waiguan*; LI4, *Hegu*; GB33, *Xiyangguan*; GB34, *Yanglingquan*; ST36, *Zusanli*; ST37, *Shangjuxu*; GB39, *Xuanzhong*; LR3, *Taichong*; KI3, *Taixi*; SP6, *Sanyinjiao*; SP10, *Xuehai*; EX-UE9, *Baxie*; NSCs, neural stem cells; NGF, nerve growth factor; BDNF, brain-derived neurotrophic factor; GAP-43, growth associated protein-43; SYN, synaptophysin; NR1, N-methyl-d-aspartate receptor subtype 1; TRPV1, transient receptor potential vanilloid subtype 1; PSD-95, postsynaptic density-95; LTP, long-term potentiation.

In conclusion, spontaneous recovery takes place in the damaged brain due to neuroplasticity after ischemic stroke. However, the recovery is often incomplete, eventually leading to neurological deficit. Several lines of clinical evidence have shown that acupuncture can improve neurological deficits induced by ischemic stroke, particularly dyskinesia, spasticity, cognitive impairment, and dysphagia. Acupuncture plays a key role in regulating neuroplasticity, as shown in Figure 2. Specifically, acupuncture promotes the replacement of functional cells that have died after stroke by stimulating nerve cell regeneration, laying the structural foundation for the establishment of contacts and synapses between the cells by promoting the growth of new nerve processes or the regeneration of damaged axons, and thus ultimately promoting information transfer by enhancing structural connections and transmission by individual synapses, neural networks, and brain regions, leading to neuroplasticity-mediated structural reconstruction and functional recovery. Glial cells represented by astrocytes and microglia, as well as the NTFs secreted by them, such as BDNF and NGF, play important roles in supporting and promoting the neuroplasticity regulated by acupuncture. However, many factors have not been discussed in depth in this review due to the lack of direct evidence, for example, there are few studies on the role of M2 microglia in acupuncture-regulated post-stroke neural plasticity, which will be the focus of future research. In addition, high-quality clinical trials and systematic investigation of relevant mechanisms are needed to obtain more conclusive evidence. For example, while it has been found in clinical studies that many nuclei are activated after acupuncture, the conduction paths of the neural circuits require confirmation by systematic basic research. In addition, the compatibility of acupoints, dose-effect relationships, time windows of intervention, and other parameters, require in-depth investigation to promote a greater clinical application of acupuncture in the treatment of ischemic stroke.

## Author Contributions

ZX and YaG: conceptualization. SQ and ZZ: methodology, data collection, and manuscript writing. YZ and JL: data collection and analysis. YGo and WF: preparation of the figures and the graphical abstract. YoG, YiG, JQ, ZX, and YaG: review and editing. All authors contributed to the article and approved the submitted version.

## Funding

This study was financially supported by the National Natural Science Foundation of China (NSFC) No. 82004464, 81904295, and 81873369; The Natural Science Foundation of Tianjin No. 20JCQNJC00920.

## Figures and Tables

**Table:** 

2VO	2-vessel occlusion model
Akt	protein-serine-threonine kinase
BDNF	brain-derived neurotrophic factor
BI	Barthel Index
BrdU	5-bromo-2’-dexoyuridine
CNS	central nervous system
CREB	cyclic adenylate monophosphate response element-binding protein
CST	corticospinal tract
CXCR4	C-X-C chemokine receptor 4
EA	electroacupuncture
ERK	extracellular signal-regulated kinase
F-actin	Filamentous actin
FMI	Fugl-Meyer Assessment scale
FOXG1	forkhead box protein G1
GAP-43	growth associated protein-43
GDNF	glia cell line-derived neurotrophic factor
GFAP	glial fibrillary acidic protein
GSK3	glycogen synthase kinase 3
IFN-γ	interferon-gamma
IGF	insulin-like growth factor
IL-10	interleukin-10
IL-1β	interleukin-1beta
JAK2	Janus-activated kinase 2
LIMK1	LIM domain kinase 1
LPs	superior parietal lobule
LTD	long-term depression
LTP	long-term potentiation
M1	primary motor area
MAPK	mitogen-activated protein kinase
MCAO	middle cerebral artery occlusion
miRNAs	miRs, microRNAs
MSCs	mesenchymal stem cells
NGF	nerve growth factor
NogoA	Neurite outgrowth inhibitor A
Notch1	Neurogenic locus notch homolog protein 1
NPCs	neural precursor cells
NR1	N-methyl-d-aspartate receptor subtype 1
NR4R3	nuclear receptor subfamily 4, group A, member 3
NSCs	neural stem cells
NT-4	neurotrophin-4
NTFs	neurotrophic factors
PI3K	phosphatidylinositol-3-kinase
PirB	paired immunoglobulin-like receptor B
PRG5	plasticity-related gene 5
PSD	postsynaptic density
PTEN	Phosphatase and tensin homolog deleted on chromosome 10
RA	Retinoic Acid
Rac1	RAS-related C3 botulinus toxin substrate 1
RCTs	randomized controlled trials
RhoA	Ras homolog family member A
ROCK	Rho-associated coiled-coil-containing protein kinase
SCI	spinal cord injury
SGZ	subgranular zone
SOX2	SRY-box transcription factor 2
STAT3	signal transducer and activator of transcription 3
SVZ	subventricular zone
SYN	synaptophysin
TNF-α	tumor necrosis factor alpha
TrkA	tropomyosin-related kinase A
TrkC	tropomyosin receptor kinase C
TRPV1	transient receptor potential vanilloid subtype 1
VEGF	vascular endothelial growth factor
ZNF423	zinc finger protein 423
